# Tocolytic effect of a selective FP receptor antagonist in rodent models reveals an innovative approach to the treatment of preterm labor

**DOI:** 10.1186/1471-2393-7-S1-S16

**Published:** 2007-06-01

**Authors:** André Chollet, Enrico Gillio Tos, Rocco Cirillo

**Affiliations:** 1 Merck Serono, Geneva, Switzerland; 2 LCG-RBM, I-10010 Colleretto Giacosa, Turin, Italy

## Abstract

**Background:**

Management of preterm labor by tocolysis remains an unmet medical need. Prostaglandins play a major role in regulation of uterine activity and in molecular mechanisms of human labor and parturition. There is some circumstantial evidence that prostaglandin F2α by action through the prostaglandin receptor subtype FP is effective in key events during labor uterine contraction, rupture of membranes and cervical dilation. This role of FP is briefly reviewed. In this study, we tested the hypothesis that an orally active and selective FP antagonist may arrest labor and delay parturition in animal models.

**Methods:**

We examined the effects of a small molecule selective antagonist of the FP receptor (AS604872) in inhibition of spontaneous uterine contraction in pregnant rat near term. We tested AS604872 for its ability to delay preterm birth in a mouse model in which the anti-progestin agent RU486 triggered parturition.

**Results:**

By oral or intravenous dosing AS604872 reduced markedly and dose-dependently the spontaneous uterine contractions in late-term pregnant rats at gestational days 19–21. In pregnant mice, AS604872 delayed the preterm birth caused by RU486 administration. The effect was dose-dependent with a significant increase in the mean delivery time of 16 and 33 hours at oral doses of 30 mg/kg and 100 mg/kg, respectively, in the case of labor triggered at gestational day 14. In both models AS604872 appeared more effective than the β-agonist ritodrine.

**Conclusion:**

The tocolytic activity displayed by a selective FP receptor antagonist supports a key role for the FP receptor in the pathophysiology of premature birth and demonstrates the therapeutic potential of an FP antagonist for the treatment of preterm labor cases in which uterine hyperactivity plays a dominant role.

## Background

Preterm birth is a serious health problem that remains the major cause of perinatal mortality and morbidity [1]. Spontaneous preterm labor is the major cause of premature birth. Despite important advances in our understanding of the mechanism of human parturition and the pathophysiology of preterm labor, there has been very little progress in the prediction, prevention and therapeutic management of preterm labor. Preterm increase of uterine activity is the most common expression of preterm labor. Therefore, pharmacological interventions aimed at maintaining uterine quiescence have been given much attention for the pharmaceutical management of preterm labor [2]. Tocolytic agents arrest preterm labor and prolong pregnancy up to a few days at the most but often with adverse effects on women and without clear demonstration of improvement in neonatal outcome [3,4].

Prostaglandins play a major role during pregnancy and parturition in all studied species, including human. In particular prostaglandins are involved, directly or through modulation of other endocrine or paracrine factors, in the final common pathway of preparation, activation and stimulation of uterine tissues leading to onset of labour [5-9]. Levels of prostaglandins in uterine tissues vary in time and regionally under the control of their synthesis by cyclooxygenases isozymes COX-1 and COX-2 and specific prostaglandin synthases on one hand and their metabolism by prostaglandin dehydrogenase enzymes on the other hand. Prostaglandins exert their effects through at least nine G protein-coupled receptor subtypes EP1-4, IP, FP, TP, DP, CRTH2 or DP2, some of them (EP1, EP3, FP, TP) existing as multiple splice variants [10,11]. PGE2 has dual uterotonic effects by action through EP1 and EP3 but may also exert an uterorelaxant effect through EP2 and EP4.

Prostaglandin F_2α _(PGF_2α_) contracts the myometrium *in vitro *and *in vivo *through activation of FP. Activation of FP [12] in the human myometrium by PGF_2α _results in the elevation of intracellular calcium concentration, which, in turn, leads to contraction of the uterine smooth cell muscle [13]. FP receptor protein expression increases in the rat myometrium with advancing gestational age thus enhancing sensitivity of the myometrium to PGF_2α _contractile action [14]. Likewise, FP is expressed in term human myometrium [13,15,16]. PGF_2α _has been shown to induce up-regulation of matrix metalloproteinase MMP1, an enzyme that breaks down collagen in cervical fibroblasts leading to cervical ripening [17]. PGF2α upregulates MMP-2 and MMP-9 and downregulates their naturally-occurring inhibitor TIMP-1 in human term decidua, thus accelerating the breakdown of collagen and the rupture of membranes [18]. PGF2α potentiates cortisol production by stimulating the enzyme 11β-hydroxysteroid dehydrogenase 1. Cortisol increases prostaglandin synthesis and decreases prostaglandin metabolism in human chorion trophoblasts thus creating a feed-forward loop in fetal membranes that may contribute to preterm birth [19]. Taken together all these findings strongly suggest that blocking FP activation might be beneficial for the control of preterm labour. Potentially, a tocolytic agent based on selective FP antagonism may be devoid of the side effects that were observed in the clinic with COX-1 and COX-2 inhibitors [20,21]. To test this hypothesis we have evaluated the effect of a recently identified orally active, potent and highly selective small molecule antagonist of the FP receptor (AS604872) in two rodent models of uterine contraction and preterm labor.

## Methods

### Materials

AS604872 or (2S)-3-([1,1'-biphenyl]-4-ylsulfonyl)-N-[(R)-phenyl(2-pyridinyl)methyl]-1,3-thiazolidine-2-carboxamide was chemically synthesized at the Serono Pharmaceutical Research Institute (Geneva, Switzerland). In all the in vivo experiments AS604872 was administered in NP3S (5% N-methylpyrrolidone, 25% polyethylene glycol 200, 30% polyethylene glycol 400, 20% propylene glycol, 20% saline), while ritodrine was solubilized in saline.

### *In vivo *experiments

All the in vivo experiments were performed according to the European Council Directive 86/609/EEC and the Italian Health Ministry guidelines for the care and use of experimental animals (decree 116/92).

### Uterine contraction model

Sprague Dawley CD (SD) BR non-pregnant (200–300 g) or late-term certified pregnant (19–21 gestation days; 350–400 g) female rats from Charles River, Calco, (Italy) were used. PGF_2α_- or oxytocin-induced uterine contractions in anaesthetized non-pregnant rats and spontaneous uterine contractions in anaesthetized late-term rats were all performed as described previously [22].

### Mouse preterm parturition model

Time-mated, primigravid certified pregnant CD1 mice transferred from Charles River (Calco, Italy) to our animal facility at day 11 of gestation were used. Preterm labor was induced according to previous reports with some modifications [23,24]. In detail, pregnant mice at gestational day 14 or 17 were treated subcutaneously with RU486 (mifepristone) 2.5 mg/kg/5 ml at 1:00 PM. Randomly defined subgroups of animals (n = 8–10/dose) were then treated twice daily orally with AS604872 (10-30-100 mg/kg) or ritodrine (30–100 mg/kg), or the vehicle (NP3S or saline 5 ml/kg) for a total of 4 administrations (6 PM on the same day of RU486 treatment, 8 AM and 6 PM on the next day and 8 AM on the day after). Each animal was checked for the occurrence of delivery starting from 18 hours after RU486 treatment, every three hours during the light phase, and every six hours during the dark phase. Delivery time was defined as the time of the first pup presence. The pups delivered were scored and classified as abortions, pups born alive and pups born dead. Since some of the pups may die during the time elapsing between our inspections, all the dead pups were controlled by the galenic hydrostatic pulmonary docimasy. The dead pups were dissected and a portion of the lung placed in water. Lung floating over the water indicated air content, and the ability of the pup to breathe after birth. The mean delivery time after RU486 was calculated based on the individual time of first pup delivery for each treatment.

### Statistical analysis

All data are means ± SEM. Statistical analysis was performed by one-way ANOVA, followed by Tukey's test. Statistics were performed using S-Plus2000 (MathSoft Inc., Seattle, Washington, USA) and significance assumed at the 5% level.

## Results

**Figure 1:**
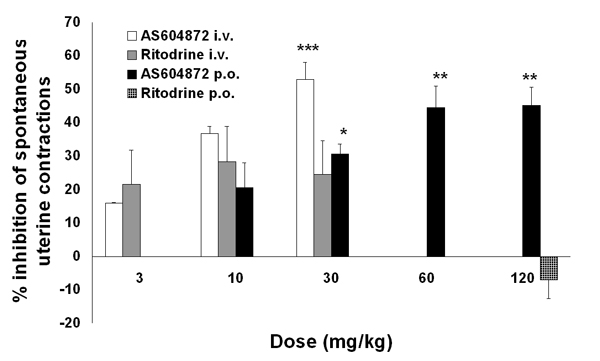
**Effects of AS604872 and ritodrine on inhibition of spontaneous uterine contractions in pregnant rats**. Anesthetized late-term pregnant rats at gestational days 19–21 were treated with AS604872 or ritodrine either by intravenous route as a 10-min infusion or orally as a single dose. The inhibition of spontaneous uterine contractions is represented in function of the dose of drugs. N = 8 animals per dose and route and drug. *p < 0.05, **p < 0.01 and ***p < 0.001 *vs *vehicle-treated group (one-way ANOVA followed by Tukey test).

The potent and selective FP antagonist AS604872 (Ki human FP, 35 nM; rat FP, 158 nM, mouse, FP 323 nM; selectivity vs EP2 50-fold and >1,000-fold vs other prostanoid receptors) reduced markedly and dose-dependently the spontaneous uterine contractions in late-term pregnant rats at gestational days (GD) 19–21 (Figure 1). Intravenous injection at the two highest doses (1 and 3 mg/kg/min for 10 min infusion corresponding to a total dose of 10 and 30 mg/kg, respectively) inhibited uterine contractility by about 37 and 53%, respectively. By oral dosing, AS604872 was also able to reduce the spontaneous uterine contractions by around 28% and 32% of the pre-dose value at doses of 30 and 60 mg/kg, respectively (Figure 1). The inhibiting effect by oral route peaked at about 30 min after administration and remained at a sustained level up to the end of the observation period of 3 h (data not shown). The intravenous administration of ritodrine decreased the spontaneous uterine motility by about 25% without a dose-dependent relationship, whereas a dose of 120 mg/kg by oral route was ineffective (Figure 1).

**Figure 2:**
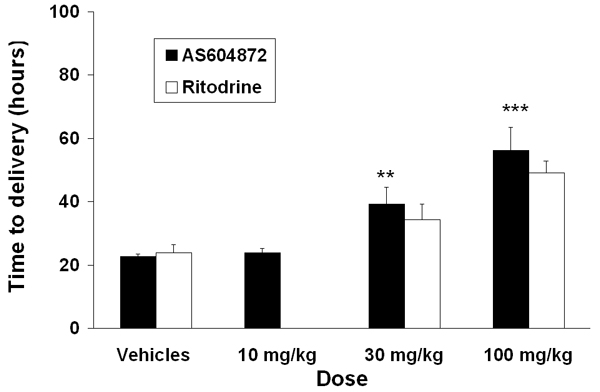
**Delaying effect of AS604872 and ritodrine on preterm parturition in mice induced at GD14**. Preterm parturition was induced by subcutaneous treatment with RU486 (mifepristone) at GD 14. Then different doses of AS604872 or ritodrine were administered orally b.i.d. and time to delivery was monitored (see Methods). Data are expressed as mean time to delivery ± SEM from the induction, n = 10.

**Figure 3:**
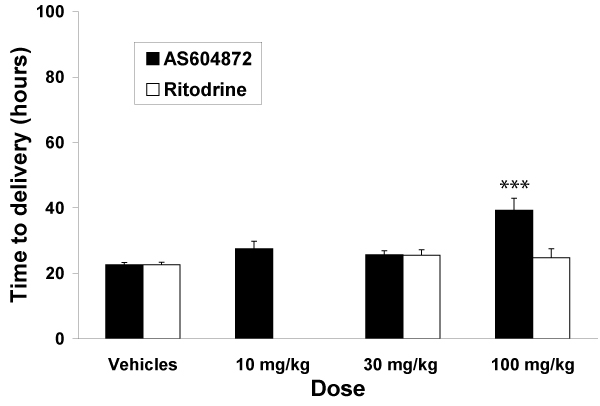
**Delaying effect of AS604872 and ritodrine on preterm parturition in mice induced at GD17**. Preterm parturition was induced by subcutaneous treatment with RU486 (mifepristone) at GD 17. Then different doses of AS604872 or ritodrine were administered orally b.i.d. and time to delivery was monitored (see Methods). Data are expressed as mean time to delivery ± SEM from the induction, n = 10.

Oral treatment with AS604872 delayed the preterm birth caused by RU486 (mifepristone) administration in pregnant mice at GD 14 or GD17. The effect was dose-dependent with a significant increase in the mean delivery time of 16 and 33 hours at doses of 30 mg/kg and 100 mg/kg, respectively, in the case of labor triggered at GD14 (Figure 2). The parturition retardation effect of AS604872 on labor induced at GD17 was less marked with a delay of 16 hours at 100 mg/kg (Figure 3). In this model AS604872 was more effective than ritodrine at GD17 whereas both compounds were comparable at GD14.

**Figure 4:**
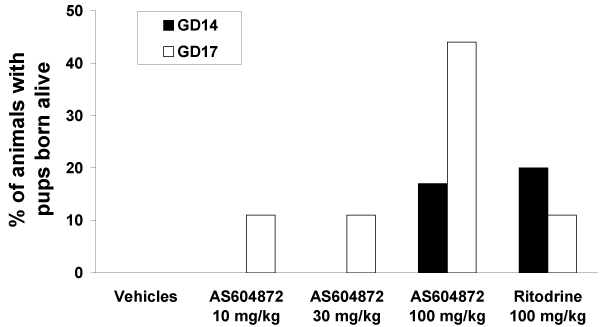
**Effects of AS604872 and ritodrine on viability of preterm mouse pup**. Data are expressed as % of dams that delivered live pups following labor induction by RU486 at GD14 or GD17 and treatment with different oral doses of AS604872 or ritodrine. The mean total number of pups (abortion, born dead and born alive) for each treatment group (9–11) was not statistically different.

In the RU486 preterm parturition model, as an important consequence of the prolongation of gestation, some dams treated with AS604872 or ritodrine delivered viable pups (Figure 4). The proportion of live pups was higher in the GD17 than in the GD14 model. Remarkably, when labor was induced at GD17, 44% of the 100 mg/kg AS604872 treated animals delivered viable pups compared to only 11% in the group treated with 100 mg/kg ritodrine. All the other deliveries resulted in abortions.

## Discussion

We have used a potent and selective small molecule antagonist of the FP receptor to decipher the role of this prostanoid receptor subtype in preterm labor and parturition. It was known that FP-deficient mice showed impaired parturition by lack of luteolysis and consequently absence of progesterone withdrawal, a necessary event to trigger labor in most animal species [25]. One key finding of our study was that blocking FP had also a clear effect on the myometrium through inhibition of uterotonic pathways in the pregnant rat model of uterine contractility. It is interesting to note that uterine activity was not fully abolished by treatment with AS604872, suggesting that other biochemical pathways are likely to be activated towards term and underlying the complex pathophysiology of preterm delivery. In the mouse parturition model, the induction of labor by the anti-progestin agent mifepristone (RU486) activates endocrine pathways and a drop in progesterone level characterized by the up-regulation of labor-associated proteins as seen in the case of idiopathic preterm labor. The FP antagonist AS604872 delayed parturition triggered by RU486 thus indicating that FP plays a central role in preterm labor of endocrine etiology. There was a trend towards better tocolytic efficacy of AS604872 at GD14 compared to GD17. This may suggest that other biochemical pathways to labor and parturition are activated at advanced gestational age. One key observation was the enhanced viability of pups delivered by mice treated with AS604872, thus underlying the beneficial effect of prolonging gestation. In both models, AS604872 was benchmarked against the β-mimetic ritodrine that has been commonly used as tocolytic agent. There was a clear trend towards superior activity for AS604872.

Our findings support recent reports that used a non-competitive, non-selective and injectable FP peptide antagonist in sheep and human myometrial strips [26,27].

## Conclusion

The tocolytic activity displayed by the selective antagonist AS604872 supports a key role for the FP receptor in the pathophysiology of premature birth and demonstrates the therapeutic potential of an FP antagonist for the treatment of preterm labor cases in which uterine hyperactivity plays a dominant role.

## List of abbreviations

FP, prostaglandin F2α receptor; COX-1, COX-2, cyclooxygenase 1 or 2; PGF2α, prostaglandin F2α; GD, gestation day; MMP, matrix metallo-proteinase; TIMP, tissue inhibitor of matrix metallo-proteinase.

## Competing interests

The authors declare that they have no competing interests.

## Authors' contributions

AC was involved in the study design, data analysis and interpretation, and drafted the manuscript. EGT performed the animal experiments and was involved in data collection, analysis and interpretation. RC was involved in the design of experiments, the data analysis and interpretation. All authors read and approved the final manuscript.
